# Dataset analysis on Cu_9_S_5_ material structure and its electrochemical behavior as anode for sodium-ion batteries

**DOI:** 10.1016/j.dib.2018.08.168

**Published:** 2018-09-01

**Authors:** Mingjun Jing, Fengliang Long, Luming Jing, Xiaopei Lv, Jiaheng Zhang, Tianjing Wu

**Affiliations:** a College of Chemistry and Chemical Engineering, Hunan Institute of Science and Technology, Yueyang 414006, China; b College of Automotive Engineering, Hunan Industry Polytechnic, Changsha 410007, China; c First Senior High School of Pingdingshan, Pingdingshan 467000, China; d College of Chemistry and Chemical Engineering, Central South University, Changsha 410083, China

## Abstract

The data presented in this data article are related to the research article entitled “Facile Synthetic Strategy to Uniform Cu_9_S_5_ Embedded into Carbon: A Novel Anode for Sodium-Ion Batteries” (Jing et al., 2018) [1]. The related experiment details of pure Cu_9_S_5_ has been stated. The structure data of pure Cu_9_S_5_ and the electrochemical performance for sodium-ion batteries are described.

**Specifications Table**

**Table:** 

Subject area	*Chemistry*
More specific subject area	*Sodium-ion batteries*
Type of data	*Figure*
How data was acquired	*X-ray diffraction (XRD, Rigaku D/max 2550 VB*^*+*^*); CHI660B electrochemical analyzer (Shanghai Chenhua Instruments Co., Ltd.).*
Data format	*Analyzed*
Experimental factors	*Samples, assembled coin cells metallic sodium foil as counter and reference electrode*
Experimental features	*XRD character of sample and CV performance of electrode material*
Data source location	*Yueyang, China*
Data accessibility	*The data is with this article.*
Related research article	[1] M. Jing, F. Li, M. Chen, J. Zhang, F. Long, L. Jing, X. Lv, T. Wu, X. Ji, Facile synthetic strategy to uniform Cu_9_S_5_ embedded into carbon: A novel anode for sodium-ion batteries, J. Alloy. Compd., 762 (2018) 473–479.

**Value of the data**

• Detailed experimental data might be used in the development of further experiments in a particular area.
• The summary of material properties can be utilized to compare together and easily accessed from the various applications.
• The CV curves could be used for more scientific analysis of various metal sulfide as anode for Na-ion batteries.

## 1 Data

**Fig. 1:**
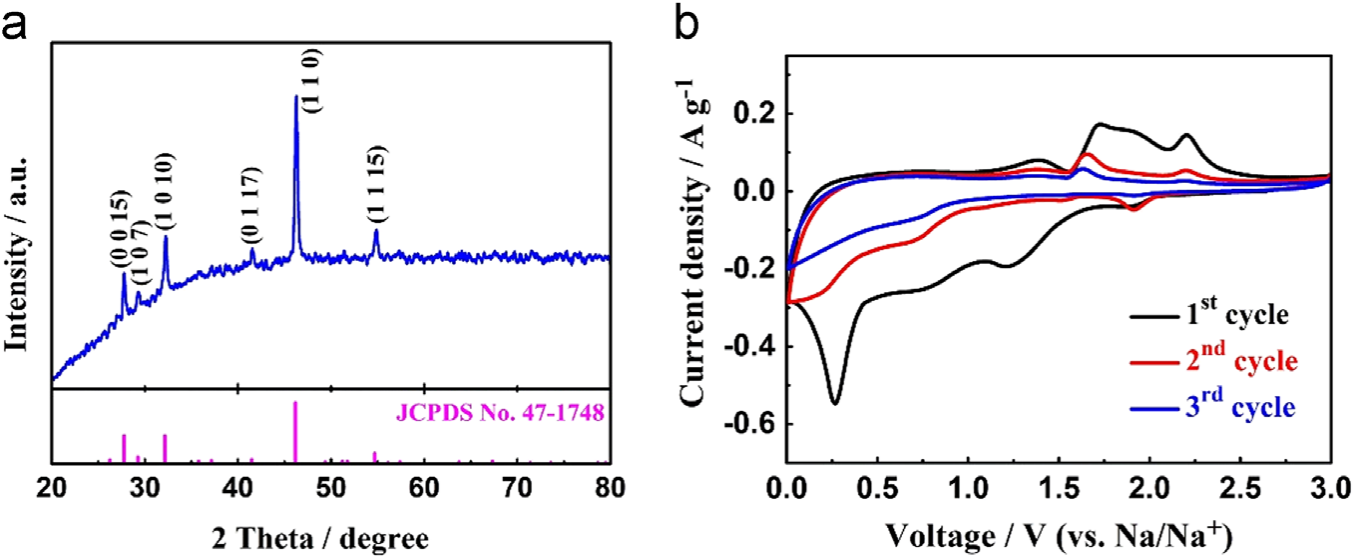
(a) XRD pattern of pure Cu_9_S_5_. (b) CV files of pure Cu_9_S_5_ electrode at 0.2 mV s^−1^.

In this data article, the detailed experimental method of pure Cu_9_S_5_ sample has been presented. The X-ray diffraction (XRD) pattern and Cyclic voltammetry (CV) files of as-prepared sample shown in Fig. 1, which is utilized to analysis the electrochemical performance of as-prepared material as anode for sodium-ion batteries.

## 2 Experimental design, materials, and methods

An ethylenediamine-assisted hydrothermal method has been utilized to prepare pure Cu_9_S_5_ nanomaterial, according to previous report [2]. The detailed experimental design is as follows. Firstly, 6.0 mL of anhydrous ethylenediamine was dissolved in 24.0 mL deionized water. 1.5 mmol of Cu(NO_3_)_2_·6H_2_O was dissolved the above solution to form a blue solution with ultrasonic dispersion for 10 minutes. And then, 1.5 mmol of thiourea was introduced to the solution in batches, while the above solution is in the process of magnetic stirring. After 30 min, the color of mixture become deep blue. Then, this mixture system was transferred into a 50 mL Teflon-lined stainless steel autoclave and treated at 120 °C for 2 h. Furthermore, black precipitates were deposited on autoclave bottom after the temperature dropping down to room temperature naturally. The pale upper clear liquid was poured out. Then, the black precipitates were washed several times utilizing absolute ethanol and distilled water with (1:1 of the volume ratio). Finally, the products were further dried at 80 °C for 24 h, and the pure Cu_9_S_5_ was obtained.

X-ray diffraction (XRD) has been utilized to investigate the crystalline structure of pure Cu_9_S_5_. The detailed test parameters are based on Ref. [1]. Cyclic voltammetry (CV) tests of pure Cu_9_S_5_ has been measured on electrochemical working station (CHI 660B) through assembled CR2025 coin cells utilizing sodium foil as counter and reference electrode, according to previous report [1].

Fig. 1a displays the XRD pattern of as-obtained Cu_9_S_5_. The diffraction peaks of material matched well to the pure hexagonal Cu_9_S_5_ phase (JCPDS card No. 47–1748). The peaks at 27.7°, 29.2°, 32.3°, 41.5°, 46.3° and 54.9° correspond to the (0 0 15), (1 0 7), (1 0 10), (0 1 17), (1 1 0) and (1 1 15) crystal planes of hexagonal Cu_9_S_5_.

Furthermore, the CV profiles of Cu_9_S_5_ were tested with the potential range from 0.01 to 3.0 V (vs Na/Na^+^) at 0.2 mV s^−1^, which is shown in Fig. 1b. In the first cathodic scan, several oxidation peaks take place from 0.2 to 2.0 V, which is mainly attributed to the oxidation reactions of Cu_9_S_5_, the insertion process of Na^+^ into electrode material, and the formation of solid electrolyte interphase (SEI) [1], [3], [4]. During the following anodic scanning, two major peaks at 1.65 and 2.12 V represent the electrochemical reaction from Cu and Na_2_S to Na_α_Cu_β_S_γ_ and further changed into Cu_9_S_5_ [5]. These obvious redox peaks of pure Cu_9_S_5_ are present, illustrating the pure Cu_9_S_5_ active material mainly presents conversion reaction in the sodiation/desodiation process. Additionally, the microstructure analysis indicates that Cu_9_S_5_ electrode material could also exhibit little insertion/extraction mechanism like other metal sulfides [6], [7].
