# Supplementary material for: Dataset analysis on Cu9S5 material structure and its electrochemical behavior as anode for sodium-ion batteries
Source: Data Brief. 2018 Sep 1;20:790–3. doi: 10.1016/j.dib.2018.08.168 (PMC6129733; doi:10.1016/j.dib.2018.08.168)
Supplement: Supplementary file 1 — Transparency document [file mmc1.docx]

**Data in Brief**

**Manuscript No.: DIB-D-18-01317**

**Conflict of interest**

All authors declared that we have no conflicts of interest to this work.

We declare that we do not have any commercial or associative interest that represents a conflict of interest in connection with the work submitted.

We understand that the corresponding author is the sole contact for editiorial process. The corresponding author signs a copyright licence on behalf of all the authors.

Signed by corresponding author: Wu Tianjing

Date: 2018-08-18
